# Excluded and myopic: Social exclusion increases temporal discounting

**DOI:** 10.1371/journal.pone.0290175

**Published:** 2023-08-15

**Authors:** Radmehr Bahrami, Khatereh Borhani

**Affiliations:** Institute for Cognitive and Brain Sciences, Shahid Beheshti University, Tehran, Iran; RIKEN CBS: RIKEN Noshinkei Kagaku Kenkyu Center, JAPAN

## Abstract

Social exclusion is a painful yet ubiquitous experience that modulates affect, behavior, and cognition. Decision-making is an essential cognitive ability that some forms of it are altered following social exclusion. However, how intertemporal decision-making is influenced by social exclusion is scarcely studied. Here, using Future Life Alone paradigm we demonstrated that experiencing social exclusion increases temporal discounting. We further tested whether the increased temporal discounting is mediated by either time perception or risk-taking. Our results revealed that although time perception is influenced by social exclusion, neither time perception nor risk-taking mediated the changes in temporal discounting. Our results are providing further evidence corroborating that social exclusion evokes cognitive deconstruction and therefore alters temporal discounting.

## Introduction

Social exclusion is the experience of being ignored or rejected emotionally or physically through social interactions [1]. As a hurting experience, exclusion threatens fundamental psychological needs such as the need to belong [2–4] and has far-reaching effects on cognition and behavior. Several studies showed decrements in intelligent thoughts [5], self-regulation [6,7], cognitive control [8], and working memory [9,10] following experiencing social exclusion.

Cognitive deconstruction is a theoretical framework that posits individuals may deconstruct their sense of self and reality in the face of traumatic experiences like social exclusion [11]. Cognitive deconstruction fortifies the self against the distress of painful realizations about the excluded self but leads to a fragmented mental state known as the cognitive deconstructed state that is characterized by decrements in cognitive functioning, particularly slower time-perception [12], and decreased self-control [13,14].

As social exclusion has been shown to have undermining effects on cognitive control and working memory, it consequently affects higher cognitive functions such as decision-making [15–17]. Studies that scrutinized the direct effects of social exclusion on decision-making are primarily concerned with aspects such as risk-taking. These studies have revealed that exclusion increases risky choices and behaviors [15–19]. However, based on preliminary studies, we speculate that in addition to risk-taking, another critical aspect of decision-making, namely temporal discounting (TD), might also be affected by social exclusion. In the current study, we were interested in examining social exclusion’s influence on TD.

TD refers to cognitive processes that involve the comparison of the value of immediate versus delayed rewards [20]. Typically, the subjective value of a delayed reward decreases as the delay in its receipt increases [21]. However, the extent to which individuals discount future rewards varies across individuals and situations [22]. TD is not a fixed trait and can be influenced by environmental factors [23,24]. The concept of TD is central to studies of impulsivity, as attaining long-term choices requires the ability and motivation to inhibit the desire for immediate gratification. High TD rates which reflects a higher tendency for immediate and smaller rewards over delayed but larger rewards have been linked to various impulse-control issues such as nicotine dependency [25–28], pathological gambling [29], and obesity [30–32]. On the other hand, low TD rates which reflect lower impulsivity, are associated with higher working memory capacity [33] and intelligence [34]. The link between TD and self-control is robust to the extent that steep TD is often deemed equivalent to impulsiveness [35].

TD is a multifaceted phenomenon that encompasses multiple cognitive and affective processes. For instance, it is sensitive to both time perception [36] and risk-taking [37], two processes that have been shown to be altered following social exclusion [12,15,16,38].

The perception of time is closely linked to impulsivity and TD [39–41]. Research shows that individuals who devalue future rewards more steeply typically tend to overestimate temporal intervals [36,40,42]. Therefore, we speculate that one mechanism that might partly explain the possible effect of social exclusion on TD could be an alteration in time perception (i.e., excluded people overestimate the passage of time and as a consequence devalue the delayed rewards more steeply). Risky decision-making is another construct whose increase leads to an increase in temporal discounting [43–46]. Hence, we speculate that risk-taking might be another candidate for explaining the effect of social exclusion on TD. However, it is important to note that recently it has been indicated that the experience of social exclusion could influence reward processing [47]. The tendency towards monetary rewards has been reported to be enhanced following exclusion, as it potentially serves as an alleviation for the pain of rejection [48]. Therefore, one might expect social exclusion to modulate TD by altering the processing of rewards within intertemporal choices.

Our study aimed to investigate the impact of social exclusion on temporal discounting. To the best of our knowledge, only one prior study has explored a related concept [12] in which the delay of gratification was examined as a subsidiary measure of time orientation by asking participants to make a decision for a friend regarding employment options with different intertemporal values. While their study touched on temporal discounting as a secondary measure, it is important to note that it was not the primary focus of their investigation. Additionally, the previous study used a hypothetical scenario to measure the delay of gratification, whereas our study used a behavioral measure that involved participants making choices with hypothetical monetary values. We also examined if social exclusion impacts time perception (in the form of interval guessing) and risk-taking (using a gambling task with explicit rules). Finally, we sought to explore whether the social exclusion directly impacts TD or whether the impact could be partly explained by an alteration in time-perception and/or risk-taking. To address these aims, the future life paradigm was used to manipulate the experience of social exclusion. Participants performed the TD task after being randomly assigned to the exclusion or inclusion groups. They then performed time perception and risk-taking tasks, presented in counter-balanced order. Affect was measured before and after manipulating the inclusionary status using the PANAS scale.

We expected to find greater TD, slower perception of time and heightened risk-taking in excluded individuals compared to included ones. We expected that time perception and risk-taking might mediate the relationship, but given that we only tested two candidate constructs, and there may be other factors at play such as reward processing, and considering the limited prior evidence on this topic, we did not have a specific hypothesis regarding the possible mediating role of time perception or risk-taking in the effect of exclusion on TD.

## Materials and methods

### Participants

The sample size was determined via a power analysis conducted in G*Power 3.1 software [49]. The social exclusion manipulation was expected to have a medium to large effect size (ηp2) of 0.3 [5,6,18,50]. The alpha was set at .05, and the power was set at 0.80. For a mixed model ANOVA, it was determined that a sample size of 28 subjects per group would suffice. Sixty neurologically healthy adult participants (30 male, *M*_age_ = 26.31, *SD*_age_ = 4.24) with normal or corrected-to-normal vision were recruited to participate in this study. They were recruited through advertising on social media and randomly assigned to exclusion (*N* = 30) or inclusion (*N* = 30) groups. Participants were informed about the procedure of the study. They gave written informed consent before the experiment and were paid equal to five dollars in compensation. Exclusion criteria were being diagnosed with psychiatric or neurological disorders, smoking more than five cigarettes per day, drinking alcohol, consuming recreational drugs, and taking painkiller medication 24 hours before the experiment. These criteria were chosen to control disease-related and drug-related effects on temporal decision-making [51]. None of the participants were excluded due to any of the mentioned criteria. The Shahid Beheshti University Ethics Committee approved the study.

### Procedure

The Participants were told that the goal is to study how intertemporal decision-making differs among personality types. The procedure began as participants filled out the consent form, a demographic form, and the Positive and Negative Affect Scale (PANAS). Next, they underwent future-life manipulation, randomly assigned to receive either inclusion or exclusion feedback. To bolster the believability of the manipulation, all participants first received real feedback regarding their scores on the Introversion/Extroversion subscale. Next, participants engaged in the TD task. Then, they completed the Game of Dice Task, and time perception task in a counterbalanced order. After finishing the tasks, participants completed the PANAS scale again to compare their affective state before and after social exclusion manipulation. They also filled out the Impressions of Personality Feedback Questionnaire (IPFQ) [52] to assess their perceived accuracy of the bogus feedback. Finally, the study’s true purpose and the necessity of inducing a sense of exclusion were explained for debriefing. Also, we made sure that everybody understood that the predictive feedback was fake and pre-written. Participants were then thanked, paid, and dismissed.

### Measures

#### Positive and Negative Affect Scale (PANAS)

PANAS [53] is a 20-item self-report questionnaire comprising two sub-scales of positive and negative affect. Each item is a word that describes an emotional and affective state. Participants rate how relatable each item is to them on a five-point Likert scale from "not at all" to "extremely." Watson et al. [53] reported a Cronbach’s alpha of 0.89 on the positive affect and 0.85 on the negative affect. In this study, we used the Farsi version of PANAS [54] with one modification. Participants were instructed to rate their current feelings instead of experienced feelings within the last month. Bakhshipour & Dezhkam [54] have reported Cronbach’s alpha of 0.87 for both the positive and the negative affect.

#### Impressions of Personality Feedback Questionnaire (IPFQ)

IPFQ [52] is a four-item questionnaire assessing the perceived accuracy and descriptiveness of the inclusionary status feedback. Participants rate the accuracy of the feedback, how much they agreed with it, how well thought-out it was, and how much a stranger could learn about them based on the feedback on a nine-point scale. Total scores range from four (least accurate and self-descriptive) to 36 (most accurate and self-descriptive). Hames et al. [52] have reported Cronbach’s alpha of 0.88 for this scale.

#### Future life paradigm

The Future Life paradigm is an inclusionary status manipulation first described by Twenge et al. [50] that elicits a sense of prolonged social exclusion [55]. The procedure begins by having the participant complete a personality test (Eysencks’ Personality Inventory) [56], and then, they are given feedback, ostensibly based on the test result. This feedback is the cornerstone of exclusion manipulation. The feedback consists of an accurate result-based description of participants’ personality type (i.e., extrovert vs. introvert), followed by a bogus prophecy about their social relations in the future. In the future alone condition (exclusion group), the participant is told that she will end up alone in future life. In contrast, in the future belonging condition (inclusion group), the participant is foretold that she will enjoy long-lasting and heartfelt relationships and friendships in the future [for the full description of feedback, see 50].

#### Temporal discounting task

In a computerized temporal discounting (TD) task [57,58] created in PsychoPy [59], participants had to choose between two hypothetical monetary rewards (i.e., a smaller-sooner reward vs. a larger-later one) in two different temporal conditions named now and not-now conditions. The not-now condition was included to demonstrate decreasing absolute sensitivity to delay [60]. The smaller-sooner reward was always available immediately in the now condition. In contrast, smaller-sooner and larger-later rewards were delayed in the not-now condition (delay of the smaller-sooner reward fixed to 60 days). Each temporal condition comprised five delay blocks: 2, 14, 30, 90, 180, and 365 days in the now condition and 62, 74, 90, 150, 240, and 425 days in the not-now condition. The presentation of blocks was counterbalanced, and participants made five consecutive choices in each block.

The larger-later reward was a fixed amount of 40 gold coins, but the smaller reward (20 gold coins at the first trial of each block) was adjusted after each trial based on participants’ previous choices. The smaller-sooner reward increased in the subsequent trial when the larger-later reward was chosen. Conversely, it decreased in the subsequent trial when the smaller-sooner reward was chosen. The first adjustment to a smaller-sooner reward was half the difference between the two rewards, and the following adjustments were half of the previous ones. These adjustments make the smaller-sooner reward converge to the point of indifference between the two rewards [61]; hence, we took the amount of the smaller-sooner reward that would have been presented in the 6th trial of a delay block as the best guess for the temporal indifference point of that delay block [22]. These indifference points can then be utilized to determine temporal indifference curves for each participant to estimate the rate at which they devalue future rewards [62]. Moreover, we included two control blocks in the TD task to assess participants’ comprehension and sensitivity to rewards. In control block 1, participants made 5 consecutive choices between 20 gold coins available immediately or 40 gold coins available immediately. The smaller reward was adjusted with the staircase procedure explained above. In control condition 2, participants chose between 20 gold coins available after 365 days and 40 gold coins available after 365 days.

We selected gold coins as the hypothetical reward since Iran (where we conducted the study) suffers from high inflation rates. This issue affects intertemporal decision-making [63] because people are disinclined to choose the larger-later reward as there is no guarantee that it is still as valuable as when they were making the decision. Since gold keeps its value over time regardless of inflation rates, it should control for the effect of inflation on intertemporal decision-making. TD rate was assessed with two measures: the TD parameter (*k*) [64], and the area under the curve (*AUC*) [65]. Using a nonlinear, least-squares algorithm, we estimated the TD parameter by fitting a hyperbolic function to the data to determine the *k* constant of the best-fitting TD function. The larger the value of *k*, the more likely participants are to select smaller-sooner rewards over larger-later ones. We estimated the area under the empirical indifference curve as an additional measure of TD that, in contrast to the TD parameter, does not depend on theoretical discounting models [65]. We first normalized subjective values and delays: subjective values were expressed as a proportion of larger-later reward (40 gold coins), and delays were expressed as a proportion of maximum delay (365 days). We then plotted the delays and subjective values on x and y coordinates to obtain the empirical indifference curve of each participant. The area under the curve is determined using the trapezoidal rule and ranges between 0 and 1. The smaller the value of AUC, the more participants are inclined to overlook the larger-later reward in favor of the smaller-sooner one.

#### Game of dice task

We designed the Game of Dice Task [GDT; 66] using Python programming language. GDT is a gambling task with explicit rules for gains and losses to assess risk-taking when no uncertainty is involved. It is presumably designed to assess the influence of cognitive control and executive functions on decision-making in a gambling scenario [67–69]. The goal is to increase the fictive money in the personal bank (initially $1000) across 18 trials by betting on a number or combination of numbers. There are four sets to choose from with different possible gains/losses. The amount of gain/loss is related to the probability of winning: the less the chance of winning, the greater the value of gains/losses. The first set includes a single number; the gain/loss is 1000 dollars. The next sets consist of combinations of 2, 3, and 4 numbers with the amount of gain/loss of 500, 200, and 100 dollars, respectively. The winning probability could be inferred readily by calculating the occurrence ratio (i.e., 1:6, 2:6, 3:6, 4:6). Therefore, the odds of win/loss are obvious. Betting on a single number or two numbers, choices with less than a 50% probability of winning, was considered risky. In contrast, betting on combinations of three or four choices with at least a 50% probability of winning was safe. Thus, the frequency of risky choices is taken as the measure of risk-taking. Additionally, we also investigated the frequency of selection of each set separately as we were interested to see if the possible impact of social exclusion would be more significant in the extreme ends of the risk-taking spectrum (i.e., choosing the highest-risk set most frequently).

#### Time perception task

We designed a time perception task based on the procedure Twenge et al. [12] described using PsychoPy software [59]. The task consisted of a warm-up and three trials. Each trial started with a picture of a traffic light that glowed in red. After a 3 seconds countdown, the light turned green for a specific time interval. The warm-up trial of 10 seconds was presented first, then intervals of 20, 40, and 80 seconds were presented in a counterbalanced order. The participants were instructed to judge for how long the light was green. Participants were not allowed to count or use other means of measuring time. Participants’ estimations were taken to measure how they perceived time flow.

### Statistical analysis

We tested the normality of data using the Shapiro-Wilk test. We used parametric measures in the case of normally distributed data (i.e., t-tests and Mixed ANOVA). When data significantly deviated from the normal distribution, we used counterpart non-parametric methods (i.e., Mann-Whitney U tests). We used two indices to determine the rate of TD (i.e., how subjective value decays as delays increase): the TD parameter [70] and the area under the empirical curve [65].

Statistical analyses and visualization were carried out using Python. We used NumPy and Pandas libraries for arranging and summarizing the data, Matplotlib and Seaborn libraries for data visualization, and SciPy.Stats and Pingouin libraries for conducting inferential statistics.

## Results

### Manipulation check

We used two measures for the manipulation check. PANAS [53] for assessing participants’ affective states before and after inclusionary status manipulation, and IPFQ [52] for assessing the perceived accuracy and descriptiveness of the inclusionary status feedback.

#### PANAS

To assess the effect of exclusion manipulation on positive and negative affective states, we ran an ANCOVA with pre-manipulation PANAS scores as the covariate, post-manipulation PANAS scores as the dependent variable, and group (inclusion/exclusion) as the between-subject variable. Results showed that exclusion manipulation significantly increased negative affect, *F*(1,58) = 6.03, *p* < .01, and decreased positive affect, *F*(1,58) = 8.19, *p* < .01 (Table 1).

**Table 1:** Means, standard deviation, and analysis of covariance for PANAS considering inclusionary status.

	Inclusion (N = 30)		Exclusion (N = 30)		*F*	*p*	*η* ^*2*^
Affect	*M*	*SD*	*M*	*SD*			
**Positive**	34.90	7.26	32.70	8.74	218.42	.001***	0.79
**Negative**	24.96	9.00	25.33	9.18	210.25	.001***	0.78

*Note*. Pre-scores are used as covariates. PANAS = Positive and negative affect scale.

***p < .001.

#### IPFQ

We used IPFQ to assess the perceived accuracy and descriptiveness of the inclusionary status feedback. Scores ranged from 4 (least credible) to 36 (most credible). The IPFQ mean score for included and excluded participants were 28.4 (*SD* = 4.85) and 27.16 (*SD* = 7.25), respectively. Mann-Whitney test showed that the groups did not differ significantly from each other in perceiving inclusionary status feedback (*p =* .460). These results indicate that the manipulation’s feedback was reasonably credible, and participants perceived it as self-descriptive regardless of the positivity or negativity of the feedback.

### Temporal discounting

**Fig 1:**
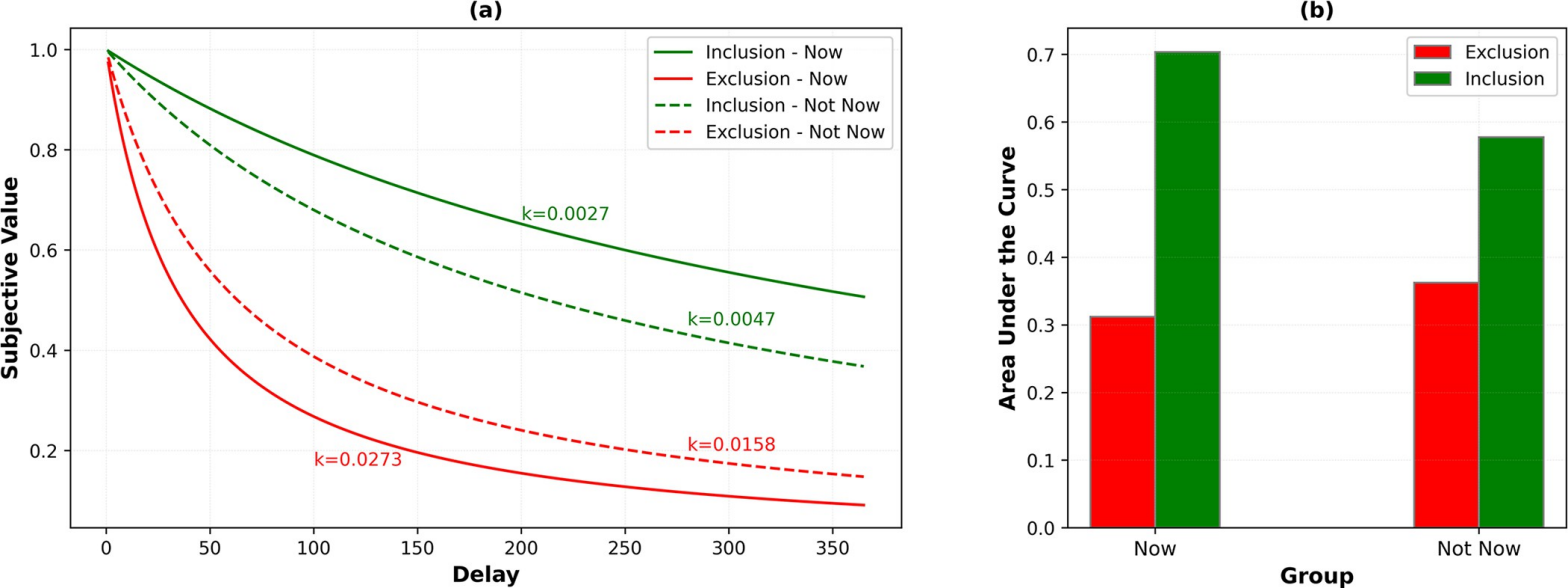
Temporal discounting curves. (a) Temporal discounting curves by participant groups (exclusion/inclusion) and delay in different temporal conditions (now/not-now). The hyperbolic curves show the discounting of subjective value as a function of time. (b) The area under the discounting curve by participant groups (exclusion/inclusion) and in different temporal conditions (now/not-now).

Fig 1A shows the TD curves by participant groups (exclusion/inclusion) and delay in different temporal conditions (now/not-now). The *k* value for each curve represents the geometric mean of the group. Since the distribution of *k* values is positively skewed, the geometric mean provides a better measure of central tendency. Excluded participants discounted future rewards more steeply than included ones in the now (geometric mean of *k*: .027 vs. .003) and not-now conditions (geometric mean of *k*: .016 vs. .004). In both control conditions, all of the participants always chose the larger reward over the smaller reward, expressing sufficient comprehension, reward sensitivity, and attention to the task. Fig 1B shows the AUC by participant group and temporal condition. Overall, Fig 1 suggests that excluded participants favored the smaller-sooner reward in both temporal conditions. We tested these impressions using a Mixed ANOVA analysis.

#### k

**Fig 2:**
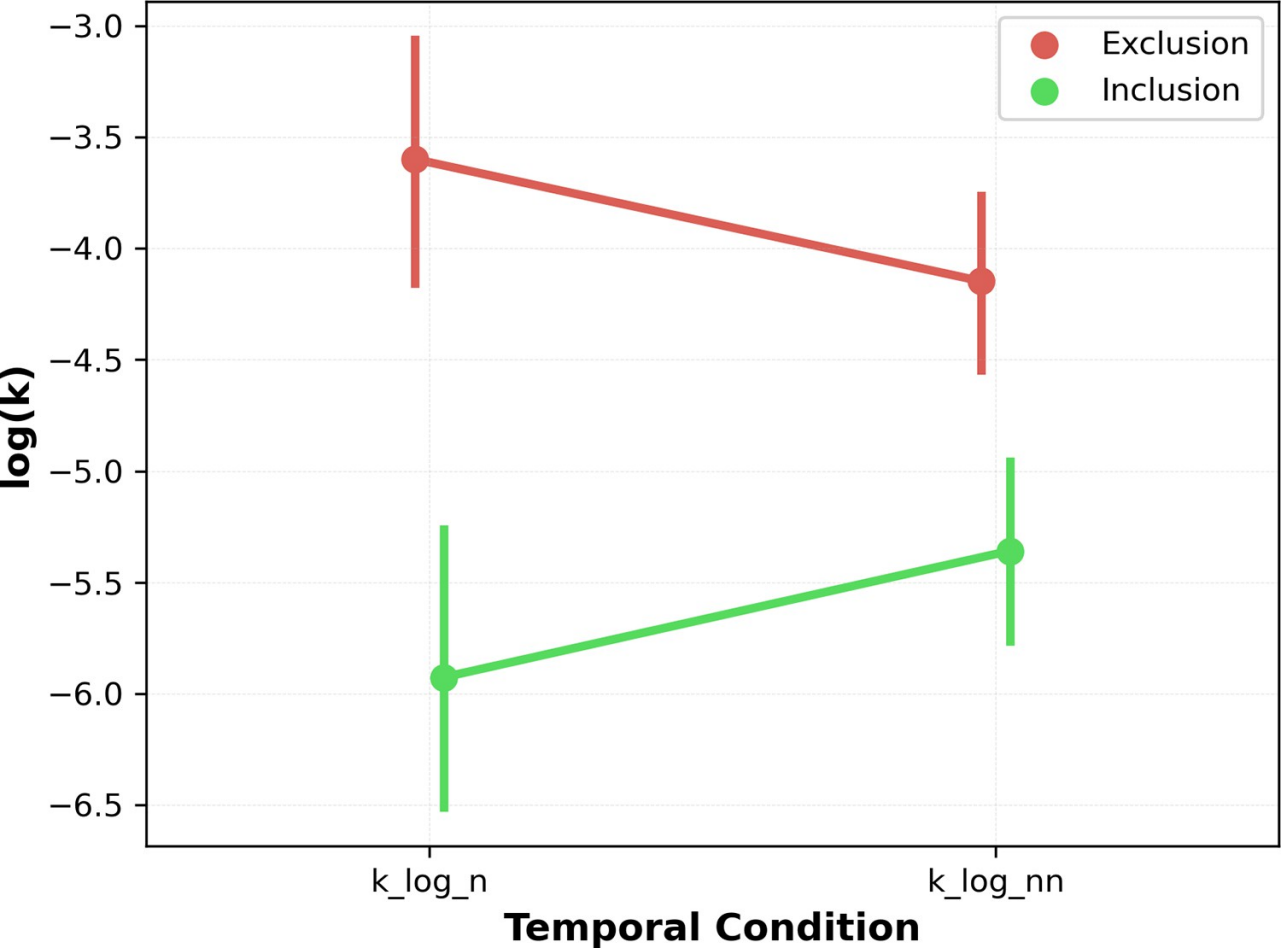
Hyperbolic rate of discounting in different exclusionary groups and time points. The difference between log-transformed *k* values in exclusion and inclusion groups in the now and not-now conditions.

An ANOVA on log-transformed *k* values with Group (inclusion, exclusion) as a between-subject factor and Temporal condition (now, not-now) as a within-subject factor yielded a significant effect of Group, *F*(1,58) = 26.22, *p* < .001, where excluded participants discounted delayed rewards more steeply (-3.87 vs -5.63; *p* < .01). The main effect of Temporal condition was not significant, *F*(1,59) = 0.005, *p* = .93. Furthermore, there was a significant Group × Temporal condition interaction, *F*(1, 1) = 18.13, *p* < .001. Post hoc Bonferroni-corrected t-tests revealed that, among the excluded participants, the discounting of future rewards was significantly greater in the now condition compared to the not-now condition (*t*(29) = 3.09, *p* < .01). In contrast, among the included participants, discounting was significantly greater in the not-now condition compared to the now condition (*t*(29) = -2.94, *p* = .01). Fig 2 depicts the interaction between groups and temporal conditions.

#### AUC

Similar results were obtained by an ANOVA on log-transformed AUC scores as the dependent variable with Group (inclusion, exclusion) as a between-subject factor and Temporal condition (now, not-now) as a within-subject factor. There was a significant effect of group, *F*(1,58) = 28.65, *p* < .001, where excluded participants discounted delayed rewards more steeply (-1.32 vs -0.52; *p* < .01). There was no significant effect of temporal condition, *F*(1,59) = 0.28, *p* = .59, and a significant Group × Temporal condition interaction, *F*(1,1) = 17.01, *p* < .001. Post-hoc analysis using pairwise t-tests showed that the difference between log-transformed AUC scores in exclusion and inclusion groups was greater in the now condition, *t*(58) = -5.83, *p* < .001, compared to the not-now condition, *t*(58) = -3.82, *p* < .001.

### Risk-taking

Out of the 18 attempts, participants in the inclusion group made slightly more safe decisions compared to the exclusion group, 11.19 (*SD = 5*.*69) vs* 10.48 (*SD = 4*.*8)*. Similarly, participants in the exclusion group made slightly more risky decisions compared to the inclusion group, 7.57 (*SD* = 4.8*) vs* 6.8 (*SD* = 5.69). However, The Mann-Whitney *U* test on GDT scores revealed that this difference is not statistically significant, *U* (58) = 485.0, *p* = 0.60. We took the final bank amount as a measure of how well participants performed the task (to maximize the fictive money in the bank). Both groups underperformed in the gambling task, and inclusion and exclusion groups ended up with a mean final bank amount of -1654.83 (*SD =* 3579.55) and -1627.576 (*SD* = 2576.11) dollars, respectively. The details of GDT measures in each group is presented in Table 2.

**Table 2:** Means, standard deviation, and Mann-Whitney U test on GDT scores based on frequency of each choice.

Variable	Exclusion (*N* = 30)		Inclusion (*N* = 30)		*U*	*P*
	*M*	*SD*	*M*	*SD*		
**Frequency of riskiest choice**	3.03	3.05	3.03	4.68	***502***.0	0.43
**Frequency of second risky choice**	4.48	3.35	3.77	3.55	512.5	0.35
**Frequency of second safe choice**	5.44	3.69	5.0	4.21	495.0	0.50
**Frequency of safest choice**	5.03	4.79	6.19	5.09	389.5	0.37
**Sum frequency of risky choices**	7.51	4.8	6.80	5.69	485.0	0.60
**Sum frequency of safe choices**	10.48	4.8	11.19	5.69	414.0	0.60
**Final bank amount**	-1627.57	2576.11	-1654.83	3579.55	420.0	0.66

*Note*. Probability of win for each choice: Riskiest = 16%, fairly risky = 33%, fairly safe = 50%, safest = 66%; initial bank amount = 1000$; total number of choices = 18.

### Time perception

A *t*-test was used to compare the mean time estimation for the 20s, 40s, and 80s intervals. For all the test trials, individuals in the exclusion group significantly estimated the interval to be longer than included participants (all *p*s ≤ .02). For the 20s interval, the time estimation of the exclusion group (*M* = 21, *SD* = 4.17) compared to the inclusion group (*M* = 18.4, *SD* = 4.3) was significantly longer, *t* (58) = 2.23, *p* = .02. Results were similar for the 40s and 80s intervals. In the 40s interval, the mean of the time estimation for exclusion and inclusion groups was 42.93 (*SD* = 9.44) and 35.3 (*SD* = 9.79) respectively, *t* (58) = 3.07, *p* < .01. Finally, in the 80s interval, the exclusion group (*M* = 80.23, *SD* = 14.28) compared to inclusion group (*M* = 65.23, *SD* = 15.43) estimated the time significantly longer, *t* (58) = 3.90, *p* < .01.

### Investigating the possible mediatory role of risk-taking and time perception for temporal discounting

We conducted mediation analysis using a bias-correct nonparametric bootstrap method with social exclusion as the independent variable, log-transformed TD rates in the now condition as the dependent variable, and frequency of risky choices and mean of interval predictions as mediator variables. There was a significant direct effect of social exclusion on TD (ß = 2.1, SE = 0.32, 95% CI 1.46 to 2.74), whereas no significant indirect effect was found for time perception (ß = 0.04, SE = 0.08, 95% CI -0.08 to 0.24) nor for risky decision-making (ß = 0.03, SE = 0.06, 95% CI -0.02 to 0.26). These results were consistent regardless of the choice of TD-dependent variable (e.g., TD rates in not-now condition, AUC in now and now-now conditions).

## Discussion

Prior research has shown that social exclusion has far-reaching impacts on cognition. Decision-making is one of the cognitive capabilities that is affected by social exclusion [15,71]. Despite several studies exploring how social exclusion affects some aspects of decision-making, such as risk-taking [15,16,38], no prior study has directly examined the impact of social exclusion on intertemporal decision-making. In the present work, we sought to study how social exclusion affects intertemporal choice in the context of TD. We also investigated the interplay between TD, risk-taking, and time perception in the aftermath of social exclusion. We used Future Life Paradigm [50] to manipulate participants’ sense of belonging or exclusion and assessed the effectiveness of inclusionary status manipulation via two measures of self-reported feedback credibility [52] and change in affective status [72].

Our results indicate that social exclusion leads to excess TD, regardless of temporal condition (i.e., the smaller-sooner reward available immediately vs. after a fixed delay). Excluded participants discounted future rewards more steeply than included ones in both temporal conditions. The excluded participants exhibited discount rates 10.248 times higher than those included, based on geometric means in the now condition. To better illustrate the stark difference, the subjective value of a unit of reward after a 50 days delay is approximately 0.90 in the inclusion group, while the subjective value at the same delay in the exclusion group is approximately 0.40. This means that the subjective value of a reward delayed by 50 days loses ~ 10 percent of its value for a person in the inclusion group and ~ 60 percent of its value in the exclusion group. Similarly, in the not-now condition, the discount rate in the exclusion group is 3.358 times higher than in the inclusion group. While the difference is still statistically significant, the decline in the ratio in the not-now condition might suggest that participants are less inclined to choose the smaller-sooner reward when both rewards are delayed (a phenomenon known as preference reversal). Moreover, excluded participants showed a significant increase in discounting future rewards in the now-condition compared to the not-now-condition, while included participants exhibited the opposite pattern. Since the groups did not differ on demographical measures such as age and sex, these results suggest that the effect of temporal framing on discounting behavior may depend on the inclusionary status of participants.

Social exclusion also reduces meaningful (intelligent) thought [5]. This condition may occur after the cognitive deconstructed state because a prerequisite for meaningful thought is the ability to encompass an extended time in the mind [73], and social exclusion nails the temporal orientation to the present moment, effectively confining the capability of mental time travel and hindering intelligent thought.

Moreover, temporal orientation is associated with the degree of TD. For instance, altering temporal orientation in favor of future gains reduces TD [74]. Since social exclusion amplifies present orientation and diminishes meaningful thought [5,12,75], the motive or ability to delay gratification could also degenerate and this is revealed by greater TD in our exclusion group.

Prior to our study, the hypothesis of intertemporal decision-making being influenced by exclusion was tested once by asking the participants to advise a friend about two career options: one with a high starting salary without a prospect of raise and another with a low starting salary but the prospect of substantial raise in a few years. Excluded participants favored the option with a higher initial salary, indicating a proclivity for immediate gratification [12]. While these results suggest that social exclusion may lead to higher TD rates and a preference for immediate rewards, which is in line with the findings of current study, the evidence is limited. The study asked participants a single question about two hypothetical job options to infer their temporal preferences. However, temporal discounting is a complex psychological process that is best measured using a systematic approach. A single choice between two options cannot adequately capture an individual’s TD profile or precisely quantify how it may have changed due to social exclusion. Moreover, in the mentioned study participants are asked to advise a friend, hence they are not concerned with facing the outcome of their decisions, whereas is our study we directly measured the impact of social exclusion on intertemporal choice on a personal level.As the cognitive deconstruction theory [11] suggests, excluded individuals deliberately try to escape self-awareness to mitigate the painful realization of their threatened belonging needs [55,76]. However, suppressing self-awareness is entwined with decrements in cognitive capabilities namely time perception and self-control [12]. Our results align and corroborate to these two features of the deconstructed state as they are crucially involved in the TD process. Furthermore, since TD is considered a measure of impulsivity, our findings are consistent with predictions that social exclusion impairs self-regulation [6,77] and causes self-defeating behavior [18,75,78].

Our results showed that socially excluded participants perceived the passage of time as slower than included participants. The perception of time is a crucial component in decision-making [29,36,79,80] and impulsive individuals perceive the passage of time more slowly [41,42]. It has been suggested that the overall level of time contraction (i.e., how long or short one perceives the passage of time) positively correlates to the degree of TD [81]. Excluded individuals feel stuck in the present time possibly due to escaping from self-awareness and painful anticipation of being alone in the future in our study. This is in fact, a defensive mechanism that they use to prevent themselves from thinking about the future [12,82]. Previous research has also reported that exclusion could cause people to be less future-oriented and therefore act less prosocial [83–86]. Furthermore, it has been shown that altering temporal attention to encompass future events can reduce TD [74], hence we can argue that social exclusion can increase delay discounting by fixating temporal attention into a narrow slice of time that is focused around present moment [12,87].

Alternatively, since the time estimation in our exclusion compared to the inclusion group, was closer to the exact time intervals, one might argue that the effect is driven by a positive effect of inclusion rather than exclusion. Given that individuals in the social inclusion group tended to experience more positive affect, it is possible that this feeling resembled the "flow state", leading to an underestimation of time [88,89]. However, future studies should investigate this possibility by determining the specific role of social inclusion in the flow state.

To our surprise, we did not find a significant difference in risk-taking between excluded and included participants, and our results are not consistent with the previous findings [15,16,38]. This inconsistency in results might partly be due to our paradigm for assessing risk-taking. GDT presumably assesses deliberative risk-taking processes [66,67] and social exclusion modulates cognitive control [90–93]. Excluded individuals show superiority in some aspects of cognitive control, such as conflict detection [94]. This might explain why they did not make more risky decisions than included individuals. Another possible explanation could be related to the notion that cognitive load is associated with risk-averse behavior [95,96]. As GDT presumably draws on deliberative cognitive processes [66,67], it might yield additional cognitive load upon participants’ minds. Considering that social exclusion leads to the reallocation of attentional resources [90,92,97–100], the extra cognitive load might have led to risk-averse behavior.

Moreover, the studies mentioned above all utilized the Cyberball paradigm [101] to induce social exclusion, whereas, in our study, the future life paradigm [50] was used. It has been shown that the outcomes of these two manipulation paradigms could differ from each other [76]. Consequent to experiencing exclusion by the Cyberball paradigm individuals usually attempt to regain control over their life and sense of belonging and therefore take more risks [16,38]. However, the sense of exclusion proceeded by the future life paradigm, which was used in the current study, causes numbness since it has long-lasting and severer results [102]. Importantly, previous studies have shown that severe exclusion experience which leads to diminished self-worth does not modulate risk-taking [15]. Therefore, albeit speculative, the future life paradigm might not affect risk-taking or affect it differently from Cyberball. Overall, we speculate that the contradiction in our results with previous findings might partly be rooted in research design.

Further, we found that intertemporal decision-making is directly affected by social exclusion and this effect is not mediated by time perception or risk-taking. This result indicates that social exclusion’s effect could extend to different aspects of cognition, and intertemporal decision-making is one of these aspects. Although we found that social exclusion weakens time perception, the tendency to choose the smaller-sooner rewards in excluded individuals was not completely due to the fact that they do not have a valid sense of time. Rather, exclusion had direct modulating effects on TD which is a form of decision-making. It is important to note that although time perception and temporal orientation may be considered as potentially correlated constructs [103,104], they are still separate constructs in the TD processes and both contribute to it [36,105,106]. In fact, a slower perception of time could contribute to TD different from being stuck in the present. Here, we tested the possible mediatory role of time perception and we did not find any effect, however, future research should seek whether temporal orientation, which has been reported to be influenced by exclusion, could mediate the effect of exclusion on TD.

In addition, the direct effect of social exclusion on TD revealed by the mediation analysis might have resulted from differed reward processing following being excluded. It has been indicated that exclusion increases the sensitivity to monetary reward [47], and causes excluded individuals to secure their threatened basic needs such as control by monetary reward [15]. This itself could be due to immersion in the present rather than the past or future, based on the deconstructed state hypothesis [12]. In this way, social exclusion enhances the salience of sooner and smaller rewards and diminishes the value of delayed and larger rewards.

Previous research has shown negative influences of exclusion on other forms of decision-making like risk-taking [107] and moral decision-making [108]. Here, we show the importance of previous social experiences on intertemporal decision-making. However, there are several limitations of the study. This study was conducted during the COVID-19 pandemic and there were strict restrictions on testing people in the lab. Therefore, we did not include a control group in future life paradigms alongside the exclusion and inclusion groups. When using the future life paradigm to manipulate exclusion, it is recommended to have also a control condition, providing non-social negative experience [i.g. being accident-prone; 5]. Although it has been revealed that experiencing exclusion has completely distinct and different consequences from a non-social negative injury, future research should compare the effect of such non-social experience on TD to social exclusion. Moreover, we only used the GDT as a measure of risk-taking behavior which is not an ecologically valid test for assessing risk-taking. Further studies are needed to explore the effect of social exclusion on risk-taking using other assessments like the balloon analogue test.
